# Intensive Care Unit-Specific Virtual Reality for Psychological Recovery After ICU Treatment for COVID-19; A Brief Case Report

**DOI:** 10.3389/fmed.2020.629086

**Published:** 2021-02-05

**Authors:** Johan H. Vlake, Jasper van Bommel, Merel E. Hellemons, Evert-Jan Wils, Diederik Gommers, Michel E. van Genderen

**Affiliations:** ^1^Department of Intensive Care, Erasmus MC, Rotterdam, Netherlands; ^2^Department of Intensive Care, Franciscus Gasthuis & Vlietland, Rotterdam, Netherlands; ^3^Department of Pulmonology, Erasmus MC, Rotterdam, Netherlands

**Keywords:** COVID-19, post-intensive care syndrome, critical care, virtual reality, post-traumatic stress disorder, anxiety, depression

## Abstract

A substantial number of ICU survivors are expected due to the SARS-CoV-2 outbreak, who are at risk for psychological impairments, such as post-traumatic stress disorder (PTSD), anxiety, and depression. We designed a COVID-19 intensive care unit-specific virtual reality (ICU-VR) intervention and tested it on one of our COVID-19 patients. The impact of event scale-revised and the hospital anxiety and depression scale showed that this patient suffered from PTSD, anxiety, and depression on the day of the intervention. One week after receiving ICU-VR, levels of PTSD, anxiety and depression had normalized, and stayed normalized until 6 months after discharge. In conclusion, innovative technologies, such as VR, have the potential to improve psychological rehabilitation, and should therefore be considered by clinicians for the treatment of ICU-related psychological sequelae after COVID-19.

## Introduction

In December 2019, a novel coronavirus, SARS-CoV-2, was first found in Wuhan, China, and rapidly spread around the world. The outbreak has officially been declared a pandemic by the World Health Organization in March 2020 (1). ICU-admission rates were reported as high as 16% (2). Because a majority of patients treated in the ICU for COVID-19 survive (survival rate in our ICU: 76%), a large group of ICU survivors can be anticipated.

One-third of general ICU survivors develop psychological impairments, such as post-traumatic stress disorder (PTSD), anxiety, and depression, due to their ICU stay (3, 4). These impairments are part of the post-intensive care syndrome (PICS), and cause a decrease in health-related quality of life and a patient's ability to return to their former life. Known risk factors for these impairments are prolonged mechanical ventilation, benzodiazepines, and a prolonged ICU stay (5). These factors were all present during COVID-19 ICU treatment, and clinicians must therefore be prepared for an increase in the incidence of COVID-19 stress-related psychopathological sequelae (6). Due to the large number of COVID-19 ICU-survivors, a uniform, low-time-consuming and easy-to-implement treatment modality is needed.

Virtual reality (VR) is a relatively new technique that has been proven to be effective for treating several psychological impairments, including PTSD and anxiety disorders (7–10). VR has three major advances; first, it represents a means of addressing the limitations of imaginal exposure and overcomes the inability to engage in sufficient detail and affective magnitude to recreate the traumatic event, a significant hurdle of imaginal exposure; second, it is an appropriate tool for patient information delivery, and can thus be used to provide additional treatment information, of which post-ICU patients are in need; and third, using VR, one can truthfully reconstruct phases of ICU treatment to replace and adjust possible delusional memories, the largest contributor to psychological distress (11–13). As such, Virtual Reality could be a valuable adjunct to improve psychological recovery and health-related quality of life after ICU treatment for COVID-19 (13, 14). Here, we describe the effect of an intensive care unit-specific VR intervention in the first patient that visited our COVID-19 outpatient clinic.

## Materials and Methods

**Figure 1:**
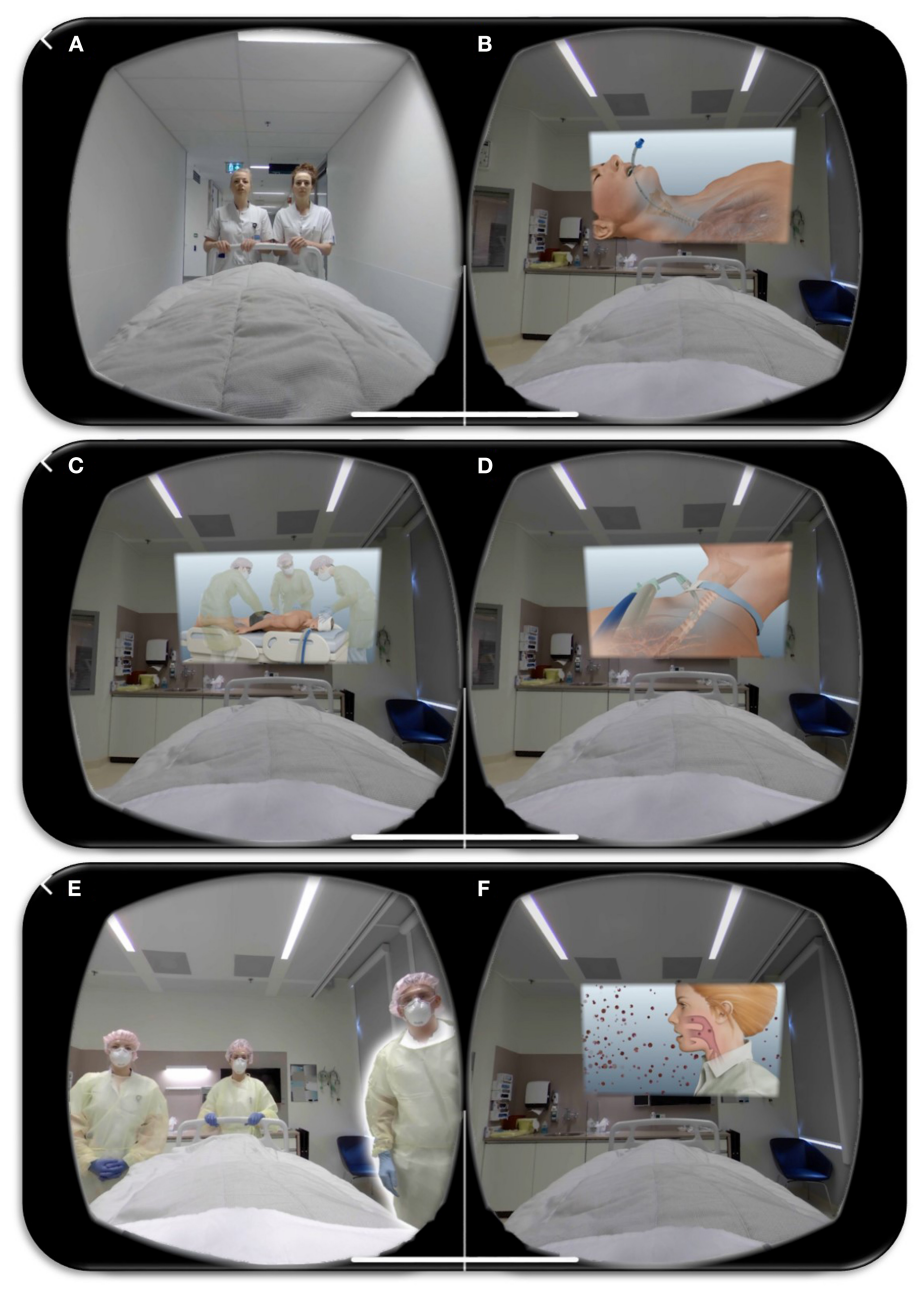
Impression of the COVID-19 Intensive Care Unit-specific Virtual Reality intervention. Screenshots of the COVID-19 Intensive Care Unit-specific Virtual Reality intervention; (1) the ICU physician and nurse welcome the patient in front of the ICU **(A)**, where the patient is virtually installed in an ICU bed. After being brought to and installed in the ICU room, explanation is given about (2) the surveillance monitor, medication pumps, intubation (including trachea tube suction) **(B)**, mechanical ventilation, and prone positioning **(C)**; (3) about the peripheral drip, central line, arterial line, gastric tube, and tracheotomy, including its procedures **(D)**; (4) about the treatment team and their responsibilities, (5) about isolation measures and personal protection equipment **(E)**, and (6) about SARS-CoV-2 and COVID-19 **(F)**.

We developed a COVID-19 intensive care unit-specific virtual reality (ICU-VR) intervention based on previous findings. A similar VR-module was tested safe in healthy volunteers and demonstrated beneficial effects in sepsis survivors (15, 16). In short, the content of ICU-VR was determined by an interdisciplinary team of ICU physicians and nurses, a psychologist, a psychiatrist, and a post-ICU patient. We adapted this module suitable for COVID-19 ICU survivors by adding additional COVID-19 specific aspects of ICU treatment (i.e., mechanical ventilation in prone position, tracheostomy and isolation measures) and information regarding SARS-CoV-2. The COVID-19 ICU-VR intervention thereby consists of six scenes; (1) the ICU physician and nurse welcome the patient in front of the ICU, where the patient is virtually installed in an ICU bed. After being brought to and installed in the ICU room, explanation is given (2) about the surveillance monitor, medication pumps, intubation (including tracheal tube suction), mechanical ventilation, and prone positioning; (3) about the peripheral drip, central line, arterial line, gastric tube, and tracheotomy, including its procedures; (4) about the treatment team and their responsibilities, (5) about isolation measures and personal protection equipment, and (6) about SARS-CoV-2 and COVID-19 (Figures 1A–F).

We tested the module in a COVID-19 patient after ICU treatment. Seven days after hospital discharge, he underwent the ICU-VR intervention twice during his visit to the outpatient clinic. After the first VR session, the patient directly asked to see the VR module for a second time. Hereafter he was given the opportunity to ask questions and to discuss the experience.

The hospital anxiety and depression scale (HADS) and impact of event scale-revised (IES-R) were used to assess anxiety-, depression-, and post-traumatic stress-related symptoms on the day of the COVID-19 outpatient clinic visit, directly prior to receiving the intervention, and 7 days and 6 months after hospital discharge (17, 18).

## Results

The patient was male, 57 years old, known with non-insulin dependent diabetes type 2, severe obstructive apnea syndrome, and no psychiatric history. He was initially admitted for respiratory insufficiency caused by a diffuse bilateral COVID-19 pneumonia, after being ill for 5 days. After 2 days of ICU treatment including deep sedation and ventilation in prone positioning, he deteriorated and was transferred to our hospital. After a consecutive treatment of 20 days in our ICU, he underwent a tracheostomy for weaning failure and started weaning in combination with intensive physiotherapy. The patient was eventually discharged from the ICU after 39 days and from the hospital after 54 days.

Seven days after discharge, he received the ICU-VR intervention twice. Prior to receiving ICU-VR, the IES-R sum score was 56, indicating severe PTSD, and the HADS depression and anxiety score were 9 and 13, indicating clinically relevant depression and anxiety, respectively. One week after receiving the COVID-19 specific ICU-VR, the IES-R score was 24, indicating none to mild PTSD, the HADS depression score was one, and the HADS anxiety score was three, indicating neither clinically relevant anxiety nor depression. More importantly, the patient stated that ICU-VR had helped him to better grasp ICU treatment, helped him to put his delusional memories into perspective, and thereby helped him to better cope with ICU-related psychological sequelae. He experienced the intervention as an enervating treatment modality and would recommend it to all patients treated for COVID-19 in the ICU.

Six months after hospital discharge, the patient suffered from critical illness polyneuropathy, had some pulmonary residual abnormalities on CT, but no decline in pulmonary function (FVC: 4.24, 82%; FEV1: 3.09, 78%). He did not suffer from psychological impairments with a still normalized IES-R score and HADS anxiety and depression scores. He partially started working again.

## Discussion

This is the first report of intensive care unit-specific virtual reality to reduce psychological distress and improve health-related quality of life after ICU treatment for COVID-19. The patient described showed a considerable decrease in post-traumatic stress disorder-, anxiety-, and depression-related symptomatology, shortly after seeing the VR module. Moreover, he reported that the intervention had helped him put his delusional memories into perspective and make sense of what happened to him during ICU treatment.

In the psychology field, VR is becoming an accepted treatment modality for several non-ICU-related psychological disorders (10). Although we are aware that the current report does not provide empirical evidence, it suggests that, especially during the current demand of care, an innovative technique, such as VR, can be considered to improve psychological well-being after ICU treatment. A larger, formally powered, randomized controlled trial should definitely demonstrate the effectiveness of our COVID-19 ICU-VR intervention. We are currently performing such a trial (Netherlands Trial Register, http://www.trialregister.nl/, identifier: NL8835).

Anxiety- and stress-related disorders are common in ICU survivors, and literature suggests that these are predominantly caused by delusional memories, sensory overload, and amnesia (19–21). A recent study by Di Nicola et al. added important knowledge and suggested a role of serum 25-hydroxyvitamine D levels and suggested a role of serum 25-hydroxyvitamine D levels in the development of symptoms of psychological stress (22). Although the role of serum 25-hydroxyvitamine D in the development of psychological sequelae after ICU treatment has not previously been examined, it could be hypothesized that low serum levels during ICU treatment could influence psychological outcomes. Future studies should examine this hypothesis, and the possibility to prevent psychological PICS by normalizing serum levels of vitamin D.

It should be acknowledged that we used self-reported questionnaires to assess the patient's psychological well-being. These questionnaires are commonly used in, and accepted for, patients after critical illness and are as such validated in critical care survivors (23, 24). One should however take into account that formal assessment of psychological disorders requires a consultation with a psychologist or psychiatrist. Additionally, although the decrease in psychological distress was substantial and can therefore be expected to be at least partially explained by the intervention, we were unable to formally rule out that other factors may have influenced the decrease in psychological sequelae.

In conclusion, innovative technologies, such as VR, have the potential to improve psychological rehabilitation, and should therefore be considered by clinicians for the treatment of ICU-related psychological sequelae after COVID-19.

## Data Availability Statement

The original contributions presented in the study are included in the article/supplementary material, further inquiries can be directed to the corresponding author/s.

## Ethics Statement

Written informed consent was obtained from the individual(s) for the publication of any potentially identifiable images or data included in this article.

## Author Contributions

JV, JvB, E-JW, and MvG designed the intensive care unit specific virtual reality film. JV, MvG, and MH recruited the patient. JV and MG wrote the first draft of the manuscript. JvB, MH, E-JW, and DG helped drafting the manuscript. All authors reviewed and approved the final version of the manuscript.

## Conflict of Interest

The authors declare that the research was conducted in the absence of any commercial or financial relationships that could be construed as a potential conflict of interest.
